# Association with Temperature Variability and Physical Activity, Sedentary Behavior, and Sleep in a Free-Living Population

**DOI:** 10.3390/ijerph182413077

**Published:** 2021-12-11

**Authors:** Jeong-Hui Park, Youngwon Kim, Gregory J. Welk, Pedro Silva, Jung-Min Lee

**Affiliations:** 1 Department of Physical Education, Kyung Hee University (Global Campus), 1732 Deokyoungdaero, Giheung-gu, Yongin-si 17014, Gyeonggi-do, Korea; jeonghee@khu.ac.kr; 2 Division of Kinesiology, School of Public Health, Li Ka Shing Faculty of Medicine, The University of Hong Kong, Pokfulam, Hong Kong, China; youngwon.kim@hku.hk; 3 Department of Kinesiology, Iowa State University, Ames, IA 50011-4008, USA; gwelk@iastate.edu; 4 CIAFEL (Research Centre in Physical Activity, Health, and Leisure), Faculty of Sports-University of Porto, 4200-450 Porto, Portugal; perrinha@gmail.com; 5 Sports Science Research Center, Kyung Hee University (Global Campus), 1732 Deokyoungdaero, Giheung-gu, Yongin-si 17014, Gyeonggi-do, Korea

**Keywords:** temperature, accelerometer, physical activity, sedentary behavior, sleep time

## Abstract

The present study examines the temperature variability in physical activity (PA), sedentary behavior (SB), and sleep in a free-living population. A representative sample of 1235 adults (ages 21–70) from Iowa, U.S.A., wore a SenseWear Mini Armband (SWA) for a randomly assigned day. Koppen’s weather climate classification was used to precisely classify the temperature: cold (−13 to 32 °F), cool (32 to 50 °F), mild (50 to 64 °F), warm (64 to 73 °F), and hot (73 to 95 °F). The main effect of three-way ANOVA (age × gender × temperature) had differences for SB and sleep, with older adults having higher levels than younger adults (*p* < 0.05). However, moderate to vigorous PA (MVPA) did not vary systematically by age or gender, and contrary to expectations, the main effect of the weather was not significant for MVPA (*p* > 0.05). Participants spent more time participating in PA at cold than at hot temperatures. The results clarify the impact of temperature on shaping PA and SB patterns in adults. The variable impacts and differential patterns by age suggest that weather should be considered when interpreting differences in PA patterns in research or surveillance applications.

## 1. Introduction

Environmental factors have received considerable attention in public health research, and there is clear evidence that they play an important role regarding participation in physical activity (PA), sedentary behavior (SB), and sleep. Chan et al. [1] demonstrated in an intervention study that variations in day-to-day activity measured by pedometer were associated with changes in the weather (i.e., temperature, precipitation, and wind speeds), as well as a day of the week and season. Temperature differences, precipitation, and sunlight exposure have frequently been reported as barriers to PA [2,3,4] and as factors influencing the amount of PA, SB, and even sleep among populations [5,6]. Weather factors are considered to have a significant influence on participation in PA [7], but a review study documented the inherent complexity of examining the relationship between weather-related factors (i.e., temperature, precipitation, and sunlight) and various activity-related variables (i.e., PA, SB, and sleep) [8]. Therefore, proving the association between season/temperature and PA, SB, and sleep time using objective measurements may affect understanding of the inherent complexity.

Leisure-time PA and physical fitness levels have generally been higher in the summer than in the winter months for people living away from the equator [9]. A study in Canada reported significant seasonal changes in PA patterns, with leisure-time PA being 86% more likely in the summer than winter. However, relationships varied across the provinces, with the weakest relationship between seasonality and PA in Newfoundland and Labrador and a stronger relationship in Saskatchewan and British Columbia [10]. A study in the UK by Sartini et al. [11] demonstrated the effects of weather on SB in older British males (71–91 years). Participants spent more time in SB (average of 26 minutes more per day) in lower temperatures (−3.5–9.2 °C) compared with higher temperatures (19.1–29.5 °C). Cepeda et al. observed complex patterns of seasonal change in middle-aged (50–64 years) and elderly (65–74 years, over 75 years) that varied in amplitude and phase by type and intensity of PA, and the time to participate in PA in summer was higher than in winter, contrary to the result of SB and sleep [12,13].

While many studies have acknowledged that weather and seasonal changes affect the time of participating in PA, SB, and sleep, other studies have had more ambiguous findings. A review by Nakashima et al. [14] reported that total time spent in MVPA did not show any differences in seasonal variations in adults, although the number of steps in winter indicated the lowest rate among four seasons. Moreover, Wang et al. [15] demonstrated no significant differences in both SB and PA (i.e., light PA and MVPA) over an entire year across different seasons. Sleep is also presumed to be influenced by seasonality; however, several studies have reported no association between seasonal and temperature changes and sleep [16,17]. Studies linking sleep with temperature variation are also unclear, perhaps due to the use of sleep diaries rather than objective measures [16,18,19]. It is likely that much of the variability in studies on temperature and seasonality can be attributed to the inevitable limitations of the measurements.

The results from previous studies tend to be inconsistent because most studies demonstrated the association between season/temperature and PA, SB, and sleep time based on subjective measurements such as questionnaires and sleep diaries rather than objective measurements. Although the association between temperature variation and activity-related variables is clearly complex, to elucidate those relations more effectively, it is critical to develop a robust design with a large and representative sample and objective measures. The present study capitalizes on data from a large observational study conducted over a two-year period to systematically evaluate differences in the intensities of PA, SB, and sleep based on temperature variability in the free-living population (free from the possible cause of diseases or chronic illnesses) in a rural Midwest US state.

## 2. Methods

### 2.1. Study Design

The study utilized data collected from the Physical Activity Measurement Survey (PAMS) project—a population-level surveillance project—conducted during 2014 and 2015. The design and protocol of the PAMS project are described in detail in a number of the primary papers on the project [20,21,22]. Briefly, using a multistage stratified sampling technique, a representative sample of adults (ages from 21 to 75) was recruited from four target counties in Iowa, USA. An initial sample of participants (purchased from Survey Sampling International) was contacted via telephone screening. This study completed the screening of 3222 households out of 5913 valid phone numbers, of which 2143 were recognized as subjects. Specifically, 1648 people completed the conceptual consent of adults to participate, and 1235 individuals, 60% of the target households, were recruited. Each county was stratified into two strata according to the proportions of Blacks and Hispanics (using tract-level demographic information from the 2000 Census). The costrata within each county were utilized to balance the distributions of Blacks and Hispanics in the sample while minimizing the variation in the sampling weights. Each participant was asked to perform both a telephone survey and a written paper survey in either English or Spanish. The protocols of the PAMS project were approved by the local institutional review board. Each participant signed written informed consent before participation.

### 2.2. Instrument

#### SenseWear Armband (SWA) Mini

The SWA Mini (BodyMedia, Inc., Pittsburgh, PA, USA) is a multisensor physical activity monitor that tracks time spent in different intensities of PA, SB, and sleep. Activity parameters are estimated using proprietary algorithms based on combined information of body movement (captured by a triaxial accelerometer) and physiological responses (assessed with heat flux, galvanic skin response, skin temperature, and near-body temperature sensors). Previous studies for validation of SWA have reported the high accuracy of the SWA for estimating energy expenditure (EE) and PA levels for adults [23,24]. The data of SWA were aggregated by ID to facilitate statistical analyses. For PAMS, the SWA was placed on the back of the upper arm, and participants were told not to wear it while showering or doing aquatic activities. Participants were asked to record activities performed while not wearing the monitor on a provided log. The SWA data were processed using the latest software version 8.1 (algorithm version 5.2).

### 2.3. Data Collection

The data for the PAMS project were collected by trained staff over two consecutive years (i.e., eight 3-month quarters). Each participant was asked to wear the SWA for 24 h on a randomly selected day, and the following day, to recall the activities performed in episodes of at least 5 min in the previous day (i.e., SWA monitoring day) through a telephone survey. The written paper survey was used to collect demographic and socioeconomic information. Specifically, each participant responded to activities of the past day from midnight to midnight in minimum bouts of 5 min across four distinct 6 h time windows (early morning, morning, afternoon, and evening). Members of the field staff went to each participant’s residence to initialize and download the monitor. The PAMS project’s design and protocols were approved by the local Institutional Review Board at Iowa State University. All participants realized enough for the study’s purpose and signed written informed consent before participation.

### 2.4. Measures

#### 2.4.1. Anthropometry

Stature was measured using the Harpenden Portable Stadiometer (Holtain Ltd., Crosswell, UK), and the values were recorded in centimeters to the nearest mm. Body mass was measured to the nearest 0.1 kg with an electronic weighing scale (Tanita Inner Scan BC 532, Middlesex, UK). Body mass index (BMI) (kg/m^2^) was calculated from the ratio of weight (kg)/height (m^2^).

#### 2.4.2. Habitual Physical Activity and Sedentary Behavior

Habitual PA and SB were assessed with the SWA for a randomly assigned day. Based on previous studies, the SWA provided valid estimates of EE and MVPA [25,26]. Also, it is particularly suitable for field-based monitoring research because it can directly measure nonwear time and have ease of use. Therefore, the current study utilized the SWA as an appropriate criterion measure to evaluate the participants’ PA, SB, and sleep. The SWA data were processed using the latest version of software v8.0 (algorithm v5.2).

#### 2.4.3. Temperature

Koppen’s weather climate classification [27,28] was used to precisely classify the temperature: cold (−13 to 32 °F, −25 to 0 °C), cool (32 to 50 °F, 0 to 9.9 °C), mild (50 to 64 °F, 9.9 to 17.9 °C), warm (64 to 73 °F, 18 to 22.9 °C), and hot (73 to 95 °F, 23 to 34.9 °C).

### 2.5. Statistical Analysis

All statistical analyses were performed by SPSS version 25 (SPSS Inc., Chicago, IL, USA), and descriptive statistics (Means and standard deviations) were used to describe the participant’s characteristics and summarize activity patterns during each month of assessment. Mean and standard deviations were calculated for PA, SB, and sleep, and *p* < 0.05 was set as the limit for statistical significance. To determine variation among gender, age, and temperature for differences in MVPA, SB, and sleep estimates, three-way mixed-model ANOVA (age × gender × temperature) and post hoc analysis was performed using Bonferroni’s correction. The least-square means and standard errors were also estimated within the model, and overall effects were examined with the standard F-test.

## 3. Results

Table 1 summarizes the sample characteristics (demographic, anthropometrics, and socioeconomic factors) across the different temperature conditions (i.e., cold, cool, mild, warm, and hot) in the area where the participants lived (*n* = 1235). The participants’ (536 males and 699 females) ages ranged between 21 and 70 years, and BMI ranged from 22.1 to 36.9, and there were no differences in BMI across the temperature codes (i.e., cold, cool, mild, warm, and hot) (*p* > 0.05). In particular, the participants’ monthly average income also varied (ranged from USD 25,000 to USD 100,000), and there were no differences between each temperature category (i.e., cold, cool, mild, warm, and hot) (*p* > 0.05).

Table 2 revealed the overall time to participate in PA-related (i.e., PA, step counts, and EE), SB, and sleep (mean ± SD) measured by objective measurement (i.e., SWA) at each different temperature. In the cold temperature range, participants spent less time participating in MVPA (M = 113.92 ± 116.04), and the number of steps indicated the lowest average (M = 8035.08 ± 5180.25). Under cool conditions (32 to 50 °F), the overall MVPA and number of steps of participants were greater (MVPA = 118.36 ± 113.75, steps = 8455.54 ± 4948.16), and SB was lower (M = 383.33 ± 232.56). In high-temperature ranges above 50 °F (i.e., mild, warm, and hot), participants showed high MVPA (mild: 124.27 ± 113.46, warm: 126.77 ± 120.89), with step counts (mild: 8897.29 ± 5301.39, warm: 10,094.75 ± 6433.82, and hot: 9622.38 ± 6436.18) and EE (mild: 2929.33 ± 826.63, warm: 3051.29 ± 926.86, and hot: 2944.67 ± 909.76) rather than at the low-temperatures (i.e., cool and cold). Although there was no difference in MVPA and EE (*p* > 0.05), the number of steps at cold and cool temperatures had significant differences from the number of steps at a warm temperature, *p* < 0.001 and *p* < 0.05, respectively.

**Figure 1:**
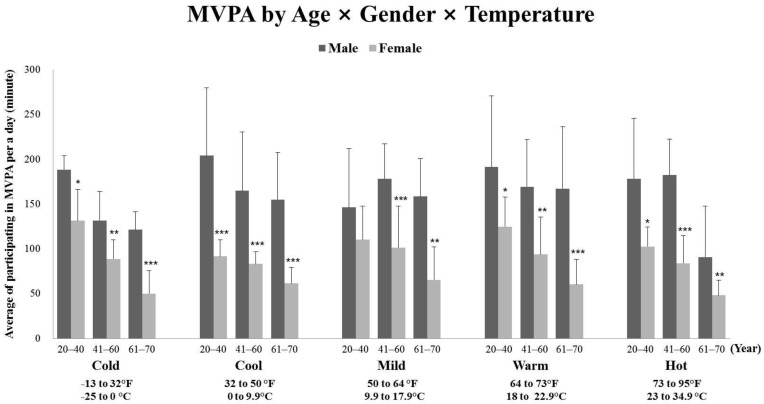
The comparisons of participating time in Moderate to Vigorous Physical Activity (MVPA) in age, gender, and temperature. *** *p* < 0.001, ** *p* < 0.01, * and *p* < 0.05. Error bars indicated the standard error of the mean (SEM).

Figure 1 indicated the mixed-model 5 × 3 × 2 (temperature × age × gender) analysis of variance (ANOVA) in MVPA. The three-way (temperature × age × gender) effect and two-way interactions approached no differences (*p* > 0.05), but there were significant main effects for the gender (males more active than females) (*p* < 0.001, *F*(1, 1205) = 118.837, partial η^2^ = 0.090) and the age group (younger groups being significantly more active than older groups) (*p* < 0.001, *F*(4, 1205) = 14.157, partial η^2^ = 0.023) by Bonferroni paired comparisons tests. 

**Figure 2:**
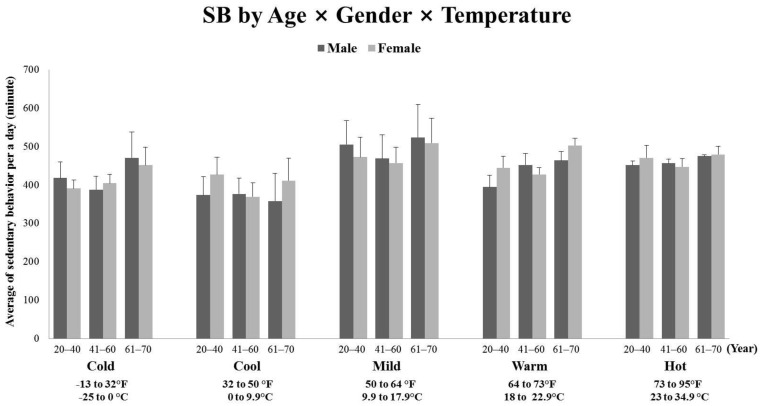
The comparisons of participating time in Sedentary Behavior (SB) in age, gender, and temperature. Error bars indicated the standard error of the mean (SEM).

Likewise, Figure 2 showed that the mixed-model 5 × 3 × 2 (temperature × age × gender) analysis of variance (ANOVA) in SB. The significant main effect for SB was temperature (*p* < 0.001, *F*(4, 1205) = 10.525, partial η^2^ = 0.034), specifically, Post hoc analyses indicated that there were strong significant pairwise differences between cool (32 to 50 °F) and mild temperature (50 to 64 °F) (*p* < 0.001) and between cool (32 to 50 °F) and hot temperature (73 to 95 °F) (*p* < 0.001). However, the effects of age group and gender were no differences for SB (*p* = 0.111 and *p* = 0.296) and three-way (temperature × age × gender) effect also had not differences (*p* = 0.824).

**Figure 3:**
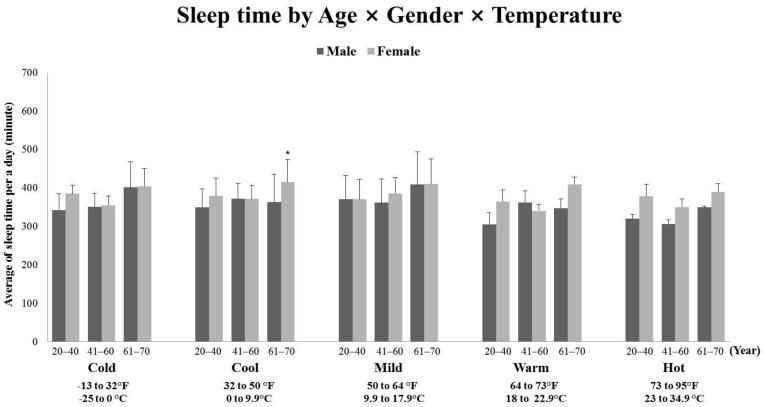
The comparisons of sleep time in age, gender, and temperature. * *p* < 0.05. Error bars indicated the standard error of the mean (SEM).

Results were different for sleep (See Figure 3) as main effects were observed for gender (*p* < 0.001, *F*(1, 1205) = 11.953, partial η^2^ = 0.010), age (*p* < 0.001, *F*(2, 1205) = 7.838, partial η^2^ = 0.013), and temperature (*p* < 0.05, *F*(4, 1205) = 2.714, partial η^2^ = 0.009). In particular, Post hoc analyses revealed that sleep time was reasonably consistent among cold (−13 to 32 °F), cool (32 to 50 °F), and warm (64 to 73 °F), but there was only evident difference between the mild (50 to 64 °F) and hot temperatures (73 to 95 °F) (*p* < 0.05). However, three-way (temperature × age × gender) effect and two-way interactions was no difference (*p* > 0.05).

## 4. Discussion

The present study examined the impact of temperature variability on PA, SB, and sleep using a large sample of adults and a robust design. The results revealed that participants had the highest levels of MVPA, steps, and EE in mild and warm temperatures. However, some differences were evident with age. Males and females had distinctly different changes in patterns of MVPA with considerably less MVPA across all temperatures (*p* < 0.001). Activity levels were higher for males compared to females, findings that are consistent with previous studies. For example, Buchowski et al. [18] measured PA using an accelerometer for seven consecutive days within one year in 57 healthy women living in the southeastern United States. They showed that women spent less time in PA, especially on weekends than males, and more time in sedentary activities. O´Connell et al. also showed that the results demonstrated that women participated less in physical activity and spent longer time sitting down rather than men, and not even participating more in winter [19].

The most interesting observations were that there was no significant difference in the time spent participating in MVPA within the same age group (*p* = 0.667) across temperatures. In fact, participants spent more time participating in Light PA, Moderate PA, and MVPA at cold temperatures (−13 to 32 °F) than at hot temperatures (73 to 95 °F). The general assumption is that low temperatures in winter would lead to less involvement in PA, but the results document that high temperatures could be more restrictive to PA than cold days. Sobolewski et al. [29] demonstrated that a hot and humid environment made the humans stay and perform a low PA because they became more exhausted when they were exposed to high ambient temperature, The temperatures exceeding 30 °C with a high relative humidity imposed upon humans an extra burden of thermal stress that added to the physical stress [30]. The longitudinal study undertaken in Perth, Australia, with weather conditions relatively constant across all seasons, showed little impact on PA behavior. Variation in weather conditions had modest explanatory power (<6%) for predicting overall and domain-specific PA engagement in this sample [31]. Therefore, the result in the current study can be explained by the differential association of temperatures with their activity preferences and patterns and discount the widely assumed notion that PA levels are lower in a cold temperature range (−13 to 32 °F).

The results also provide insights into the corresponding impact of temperature on SB. In general, participants spent less time living sedentary in cool temperatures, but patterns seemed to vary with age. Older adults (from 61 to 70 years) in both genders spent more time in SB, similar to previous studies [32,33,34,35]; however, we discovered that young-aged female participants (from 21 to 40 years) spent longer sitting down rather than middle-aged participants (from 41 to 60 years), and males also presented a similar result in cold and mild temperatures. Most middle-aged participants underwent rearrangement of their life due to retirement, so they might be provided a key opportunity for interventions to stimulate a nonsedentary and more active life. In particular, lack of time may no longer be a frequently reported obstacle to physical activity [36,37]. Barnett et al. [38] confirmed that retirement provided an opportunity to promote physical activity among middle-aged adults and that it positively impacted physical activity through increased free time and prioritization of health. Therefore, if middle-aged is a key time frame for their PA, policies in our society should be assisted in providing opportunities for them to be active, and have also acknowledged the need for indoor facilities to provide the opportunity for their year-round participation in PA [39].

The average level of sleep in the sample was six hours (total: 366.00 min), but there was considerable variability at the minutes on an individual level (cold: 367.90, cool: 376.63, mild: 382.00, warm: 355.73, and hot: 347.76). Sleep is an indispensable metabolic element for health, so sleep curtailment is related to an increased risk of not only obesity but also physiological diseases [40,41]. The National Sleep Foundation (NSF) reported recommendations of sleep duration divided into nine age categories from birth (0–3 months) through old age (≥65 years). Adults over 18 years should have 7 to 9 h (i.e., 420 to 540 min) of sleep [42]. However, according to the results of sleep, all participants have shown a lack of sleep quantity in each different temperature and age compared to the national recommendation of sleep duration, and even hot temperature showed the shortest sleep quantity in both genders (males: 325.77 and females: 372.75 min). Females spent more time sleeping compared to males, and the older adults group (from 61 to 70 years) also spent plenty of time in sleep. Furthermore, contrary to our intuitive expectation that SB and sleep would be higher at low-temperature (i.e., cold and cool), participants who lived in mild temperature spent more time sleeping rather than those who lived in cold temperature (mild: 382.00 min and cold: 367.90 min. The estimates at mild temperature were indicated as the highest rate. This result is inconsistent with previous studies. One study revealed that sleep did not show a seasonal pattern [17], and even O’Connell et al. [19] proved that moderate to vigorous physical activity and sleep among United Kingdom adults showed no significant differences in seasonal variations, although sedentary time was significantly higher in winter than in summer. It can be assumed that one of the reasons was that the temperature effect on PA had been previously studied, and the majority of studies have used subjective measures. In general, the use of methodologies that objectively quantify PA and SB variation by season complement and confirm the results primarily obtained using a questionnaire and sleep diary usually have been used as a standard sleep assessment tool.

The strengths of this study include an extensive collection of data over multiple seasons and the use of a strong monitoring device for the objective assessment of patterns. The study is the first to investigate all participants’ habitual PA, SB, and even sleep patterns across two years by using accelerometers. Our study confirmed not only the PA, SB, and sleep across temperatures but also the changes across gender and age by analysis of three-way ANOVA. Moreover, a total of 1235 adults participated in the study, which was an adequate sample size and was suitable for accepting the findings of the current study. Nevertheless, there are also a few limitations of the study. The current study had not examined indoor-related information (i.e., temperature, humidity, and their activities) at which individuals stayed, thus, subsequent studies can obtain more accurate results by controlling some factors (i.e., in/outdoor PA levels and indoor temperature) that could affect the outcomes. Also, since the study did not repeatedly measure PA, SB, and sleep of the same participant at different temperatures, it is necessary to examine more evidence with repeated measures for the variables from the same perspective. Additionally, other studies in the future need to demonstrate with various countries, races, and differences in temperature adaptation between different races the relationship between temperatures and health variables (i.e., PA, SB, and sleep). Given the nature of our study, we did not examine the associations of other indicators of weather (i.e., precipitation, clouds, and sunlight) with PA, SB, and sleep.

## 5. Conclusions

In conclusion, we demonstrated that participants showed differences in PA, SB, and sleep time (MVPA, step, and EE levels were highest at mild and warm temperatures), and the changes in MVPA patterns were different for men and women at all temperatures. Therefore, the temperature or sunlight exposure cannot be changed, but the temperature patterns suggest that weather must be considered or controlled statistically when interpreting data on PA and SB. From a public health perspective, if the temperature and weather pose specific barriers, it is also important to develop programming that takes this into account. Specifically, there is a need to provide more opportunities for indoor PA during the hot and humid periods of the year [39]. In addition, the variation in temperature in relation to levels of PA intensities and SB suggest that interventions to promote PA and decrease SB must be tailored to take into account these temperature and seasonal differences, as well as gender characteristics. The direct effect of temperature on PA also needs to be objectively assessed to better understand the effects on outdoor recreation.

## Figures and Tables

**Table 1:** Characteristics of Participants by Temperature.

Variables	*N*	Cold	Cool	Mild	Warm	Hot
		−13 to 32 °F	32 to 50 °F	50 to 64 °F	64 to 73 °F	73 to 95 °F
		(*n* = 342)	(*n* = 287)	(*n* = 208)	(*n* = 208)	(*n* = 190)
**Gender**						
Male	536	158	118	91	89	80
Female	699	184	169	117	119	110
**Ethnicity/race**						
White	1107	292	264	189	189	173
Other	128	50	23	19	19	17
**Age (year)**						
21–40	314	33.7 ± 5.4	31.2 ± 5.8	33.0 ± 5.9	34.0 ± 4.9	33.9 ± 5.2
41–60	614	51.3 ± 5.6	51.2 ± 5.6	51.7 ± 5.8	51.0 ± 5.9	50.1 ± 5.9
61–70	307	64.9 ± 3.0	65.3 ± 3.0	66.4 ± 2.4	65.2 ± 3.0	64.7 ± 3.1
**BMI (** **kg·m^−2^)**						
Normal	283	22.8 ± 1.7	22.4 ± 1.7	22.3 ± 1.9	22.2 ± 1.9	22.3 ± 1.7
Overweight	403	27.7 ± 1.4	27.6 ± 1.4	27.5 ± 1.3	27.8 ± 1.4	27.4 ± 1.4
Obese	549	36.6 ± 6.5	36.9 ±7.0	37.2 ± 6.3	36.7 ± 5.5	36.9 ± 7.1
**Current Smoking**						
Yes	231	68	61	37	30	35
No	1004	274	226	171	178	155
**Education level**						
Less than high school	49	14	13	4	9	9
High school diploma	284	82	67	49	44	42
Some college	413	108	94	70	82	59
College graduate	336	102	79	52	46	57
Graduate degree	153	36	34	33	27	23
**Married**						
Married or living as married	812	223	186	141	133	129
Divorced or separated	166	47	35	32	28	24
Widowed	51	14	14	5	12	6
Single, never married	206	58	52	30	35	31
**Income**						
less than USD 25,000	217	62	53	40	38	24
from USD 25,000 up to USD 50,000	346	99	81	51	60	55
from USD 50,000 up to USD 75,000	258	68	65	36	41	48
from USD 75,000 up to USD 100,000	195	50	44	31	37	33
more than USD 100,000	219	63	44	50	32	30

BMI: Body Mass Index, USD, A variable of ‘Other’ in Ethnicity/race included Blacks and Hispanics population; Age in years and BMI values present Mean ± Standard Deviation; All characteristics information were analyzed by descriptive statistics had no differences.

**Table 2:** Overall Physical Activity, Sedentary Behavior, and Sleep Time by Objective Measurement (SenseWear Mini Armband: SWA) (*n* = 1235).

Variables	Cold (A)	Cool (B)	Mild (C)	Warm (D)	Hot (E)	*F*-Value	Post-hoc
	−13 to 32 °F	32 to 50 °F	50 to 64 °F	64 to 73 °F	73 to 95 °F		
	*(n* = 342)	(*n* = 287)	(*n* = 208)	(*n* = 208)	(*n* = 190)		
Light PA	560.2 ± 277.9	563.6 ± 289.3	456.3 ± 242.4	516.8 ± 242.0	520.0 ± 224.6	6.7 ***	C < A, B
Moderate PA	105.2 ± 105.6	106.5 ± 102.2	112.3 ± 107.4	113.6 ± 112.0	100.8 ± 110.7	0.5	N/A
Vigorous PA	8.7 ± 20.5	11.9 ± 29.8	12.0 ± 22.2	13.2 ± 26.6	12.8 ± 36.28	1.3	N/A
MVPA	113.9 ± 116.0	118.4 ± 113.8	124.3 ± 113.5	126.8 ± 120.9	113.6 ± 126.2	0.6	N/A
Sedentary Behavior	404.3 ± 216.3	383.3 ± 232.6	481.2 ± 158.2	446.3 ± 153.9	460.3 ± 115.1	11.3 ***	B < D A, B < C, E
Sleep Time	367.9 ± 120.4	376.6 ± 122.8	382.0 ± 99.7	355.7 ± 144.9	347.8 ± 120.5	2.9 *	E < C
Steps	8035.9 ± 5180.3	8455.5 ± 4948.2	8897.3 ± 5301.4	10,094.8 ± 6433.8	9622.4 ± 6436.2	5.7 ***	A < E A, B < D
Energy Expenditure	2925.7 ± 912.3	2886.5 ± 864.0	2929.3 ± 826.6	3051.3 ± 926.9	2944.7 ± 909.8	1.1	N/A

PA: Physical Activity; MVPA: Moderate to Vigorous Physical Activity; N/A: Not Applicable; *** *p* < 0.001, * *p* < 0.05; All variables were analyzed by ANOVA.

## Data Availability

The datasets used and analyzed during the current study are available from the corresponding author on reasonable request.
